# Supplementary material for: High Disinfectant Tolerance in Pseudomonas spp. Biofilm Aids the Survival of Listeria monocytogenes
Source: Microorganisms. 2023 May 27;11(6):1414. doi: 10.3390/microorganisms11061414 (PMC10304169; doi:10.3390/microorganisms11061414)
Supplement: Supplementary file 1 [file microorganisms-11-01414-s001.zip › Supplemental Figure S1 Biofilm screening (1).pdf]

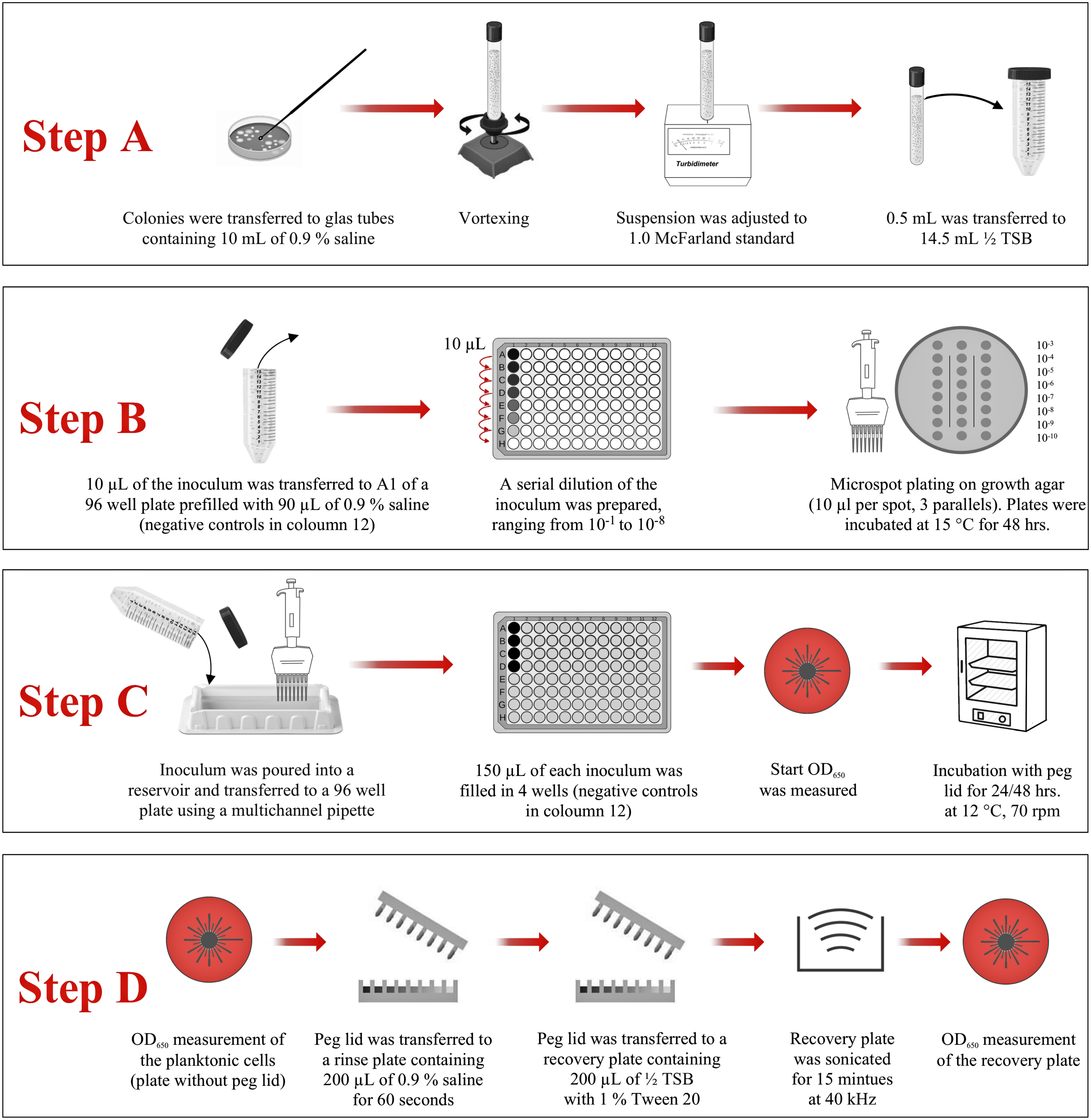

Step A
Turbidimeter
Colonies were transferred to glas tubes containing 10 mL of 0.9 % saline
Vortexing
Suspension was adjusted to 1.0 McFarland standard
0.5 mL was transferred to 14.5 mL ½ TSB
Step B
10 µL
A B C D E F G H
1 2 3 4 5 6 7 8 9 10 11 12
10^{-3} 10^{-4} 10^{-5} 10^{-6} 10^{-7} 10^{-8} 10^{-9} 10^{-10}
10 µL of the inoculum was transferred to A1 of a 96 well plate prefilled with 90 µL of 0.9 % saline (negative controls in coloumn 12)
A serial dilution of the inoculum was prepared, ranging from 10^{-1} to 10^{-8}
Microspot plating on growth agar (10 µl per spot, 3 parallels). Plates were incubated at 15 °C for 48 hrs.
Step C
A B C D E F G H
Inoculum was poured into a reservoir and transferred to a 96 well plate using a multichannel pipette
150 µL of each inoculum was filled in 4 wells (negative controls in coloumn 12)
Start OD_{650} was measured
Incubation with peg lid for 24/48 hrs. at 12 °C, 70 rpm
Step D
OD_{650} measurement of the planktonic cells (plate without peg lid)
Peg lid was transferred to a rinse plate containing 200 µL of 0.9 % saline for 60 seconds
Peg lid was transferred to a recovery plate containing 200 µL of ½ TSB with 1 % Tween 20
Recovery plate was sonicated for 15 mintues at 40 kHz
OD_{650} measurement of the recovery plate
