# Supplementary material for: High Disinfectant Tolerance in Pseudomonas spp. Biofilm Aids the Survival of Listeria monocytogenes
Source: Microorganisms. 2023 May 27;11(6):1414. doi: 10.3390/microorganisms11061414 (PMC10304169; doi:10.3390/microorganisms11061414)
Supplement: Supplementary file 1 [file microorganisms-11-01414-s001.zip › Supplemental Figure S2 MIC, MBC, MBEC, log kill (1).pdf]

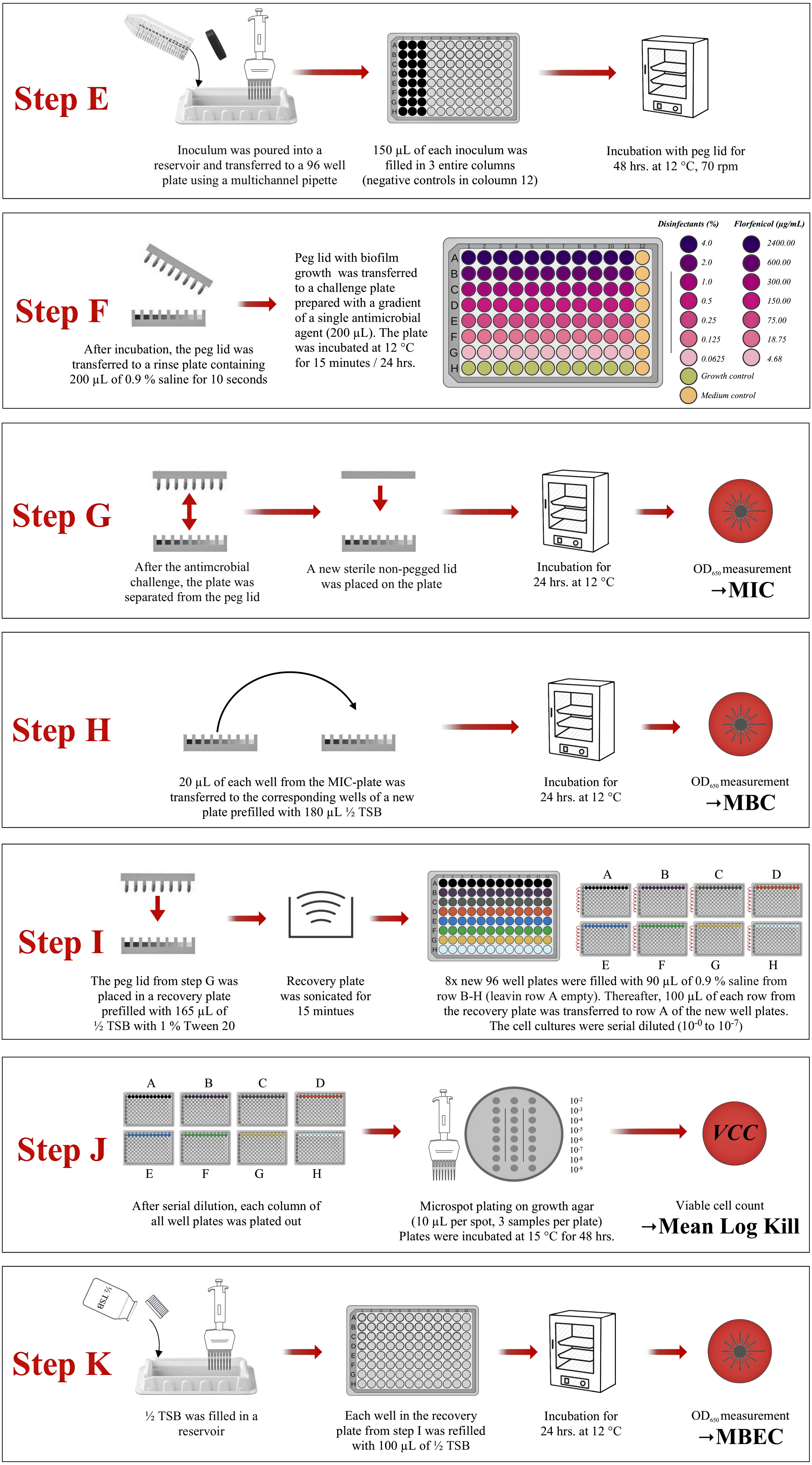
Step E
Inoculum was poured into a reservoir and transferred to a 96 well plate using a multichannel pipette
150 µL of each inoculum was filled in 3 entire columns (negative controls in coloumn 12)
Incubation with peg lid for 48 hrs. at 12 °C, 70 rpm
Step F
After incubation, the peg lid was transferred to a rinse plate containing 200 µL of 0.9 % saline for 10 seconds
Peg lid with biofilm growth was transferred to a challenge plate prepared with a gradient of a single antimicrobial agent (200 µL). The plate was incubated at 12 °C for 15 minutes / 24 hrs.
Disinfectants (%)
Florfenicol (µg/mL)
4.0
2400.00
2.0
600.00
1.0
300.00
0.5
150.00
0.25
75.00
0.125
18.75
0.0625
4.68
Growth control
Medium control
Step G
After the antimicrobial challenge, the plate was separated from the peg lid
A new sterile non-pegged lid was placed on the plate
Incubation for 24 hrs. at 12 °C
OD650 measurement
→MIC
Step H
20 µL of each well from the MIC-plate was transferred to the corresponding wells of a new plate prefilled with 180 µL ½ TSB
Incubation for 24 hrs. at 12 °C
OD650 measurement
→MBC
Step I
The peg lid from step G was placed in a recovery plate prefilled with 165 µL of ½ TSB with 1 % Tween 20
Recovery plate was sonicated for 15 mintues
A
B
C
D
E
F
G
H
8x new 96 well plates were filled with 90 µL of 0.9 % saline from row B-H (leavin row A empty). Thereafter, 100 µL of each row from the recovery plate was transferred to row A of the new well plates. The cell cultures were serial diluted (10-0 to 10-7)
Step J
A
B
C
D
E
F
G
H
After serial dilution, each column of all well plates was plated out
10-2
10-3
10-4
10-5
10-6
10-7
10-8
10-9
Microspot plating on growth agar (10 µL per spot, 3 samples per plate) Plates were incubated at 15 °C for 48 hrs.
VCC
Viable cell count
→Mean Log Kill
Step K
½ TSB
½ TSB was filled in a reservoir
Each well in the recovery plate from step I was refilled with 100 µL of ½ TSB
Incubation for 24 hrs. at 12 °C
OD650 measurement
→MBEC
